# Recognizing and monitoring infectious sources of schistosomiasis by developing deep learning models with high-resolution remote sensing images

**DOI:** 10.1186/s40249-023-01060-9

**Published:** 2023-02-07

**Authors:** Jing-Bo Xue, Shang Xia, Xin‑Yi Wang, Lu-Lu Huang, Liang-Yu Huang, Yu-Wan Hao, Li-Juan Zhang, Shi-Zhu Li

**Affiliations:** 1 National Institute of Parasitic Diseases, Chinese Center for Disease Control and Prevention (Chinese Center for Tropical Diseases Research); NHC Key Laboratory of Parasite and Vector Biology; WHO Collaborating Centre for Tropical Diseases, National Center for International Research On Tropical Diseases, Shanghai, 200025 China; 2 School of Global Health, Chinese Center for Tropical Diseases Research, Shanghai Jiao Tong University School of Medicine, Shanghai, 200025 China

**Keywords:** Deep learning, High-resolution remote sensing, Recognizing, Monitoring, Infectious source, Schistosomiasis

## Abstract

**Background:**

China is progressing towards the goal of schistosomiasis elimination, but there are still some problems, such as difficult management of infection source and snail control. This study aimed to develop deep learning models with high-resolution remote sensing images for recognizing and monitoring livestock bovine, which is an intermediate source of *Schistosoma japonicum* infection, and to evaluate the effectiveness of the models for real-world application.

**Methods:**

The dataset of livestock bovine’s spatial distribution was collected from the Chinese National Platform for Common Geospatial Information Services. The high-resolution remote sensing images were further divided into training data, test data, and validation data for model development. Two recognition models based on deep learning methods (ENVINet5 and Mask R-CNN) were developed with reference to the training datasets. The performance of the developed models was evaluated by the performance metrics of precision, recall, and F1-score.

**Results:**

A total of 50 typical image areas were selected, 1125 bovine objectives were labeled by the ENVINet5 model and 1277 bovine objectives were labeled by the Mask R-CNN model. For the ENVINet5 model, a total of 1598 records of bovine distribution were recognized. The model precision and recall were 81.9% and 80.2%, respectively. The F1 score was 0.81. For the Mask R-CNN mode, 1679 records of bovine objectives were identified. The model precision and recall were 87.3% and 85.2%, respectively. The F1 score was 0.87. When applying the developed models to real-world schistosomiasis-endemic regions, there were 63 bovine objectives in the original image, 53 records were extracted using the ENVINet5 model, and 57 records were extracted using the Mask R-CNN model. The successful recognition ratios were 84.1% and 90.5% for the respectively developed models.

**Conclusion:**

The ENVINet5 model is very feasible when the bovine distribution is low in structure with few samples. The Mask R-CNN model has a good framework design and runs highly efficiently. The livestock recognition models developed using deep learning methods with high-resolution remote sensing images accurately recognize the spatial distribution of livestock, which could enable precise control of schistosomiasis.

**Graphical Abstract:**

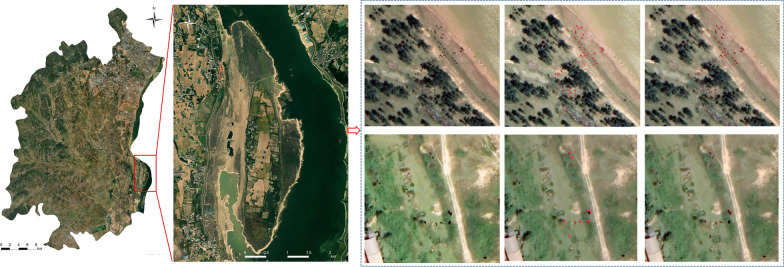

## Background

Schistosomiasis is caused by infection with parasites of the genus *Schistosoma*. Schistosomiasis is a zoonotic parasitic disease that greatly affects human health and socioeconomic development. It is one of the neglected tropical diseases identified by the World Health Organization [1, 2]. Schistosomiasis japonica was once highly prevalent in China. There were approximately 11 million human cases of schistosomiasis and 1.5 million bovines infected by *S. japonicum* at the early stage of the national schistosomiasis control program in the early 1950s in China [3]. Livestock, notable bovines, are the main source of *S. japonicum* infection, which plays a critical role in the transmission of schistosomiasis [4, 5]. Since 2004, an integrated strategy with an emphasis on controlling the source of *S. japonicum* infection has been implemented in China. A package of interventions that involve adaptions to local circumstances includes breeding livestock in fences, replacing bovines with machines, bovine removal, and prohibiting pasture in snail habitats. These interventions have proven effective in reducing the transmission of schistosomiasis [3, 6, 7]. The transmission of schistosomiasis japonica is affected by natural, ecological, and social factors, and the geographical distribution of the disease is governed by the *Oncomelania hupensis* snail, the intermediate host of *S. japonicum* [8, 9]. The number and distribution of the sources of *S. japonicum* infection determine the risk of transmission of schistosomiasis [10]. Therefore, monitoring of *O. hupensis* breeding and livestock infections has become an important part of monitoring the schistosomiasis transmission risk [11]. Because of backward agricultural production practices and the single industrial structure, bovines remain the main resource of productivity and economy, resulting in incomplete livestock removal and rebreeding following removal [4, 6]. In 2021, there were still 525,878 bovines in the schistosomiasis-endemic foci of China [12]. Dynamic monitoring and management of bovines are therefore effective in reducing the schistosomiasis transmission risk in *O. hupensis* snail-infested settings.

Recently, remote sensing has been widely used for monitoring *O. hupensis*-infested areas and evaluating the risk of schistosomiasis transmission and spread, for example, Xue et al. used high-resolution remote sensing technology to research schistosomiasis surveillance [13]. Xia et al. used multi-source remote sensing images to assess the risk of intestinal schistosomiasis transmission impacted by flooding [14]. With the increase in spatial resolution of remote sensing images and the development of computer vision technology, the visual difference between remote sensing images and natural images has gradually reduced. The acquisition of high-resolution remote sensing images has recently emerged as a viable monitoring technique for detecting wildlife. The technique has been successfully used to identify and count several wildlife species in open, homogeneous landscapes and seascapes. For example, Duporge et al. used very high-resolution satellite imagery and deep learning to detect and count African elephants in heterogeneous landscapes [15]. Laradji et al. used a deep-learning model to track illegal cattle ranching from high-resolution satellite images [16]. The benefits of this monitoring technique are numerous. For example, large spatial extents can be covered within a short time, which allows repeat surveys and reassessments over short intervals of time [17, 18]. High-resolution remote sensing images at sub-meter resolution have been effectively used for the dynamic monitoring of large animal distributions [19–21].

Previous studies have shown that the convolutional neural network (CNN) can be used to explain, extract, and classify images [22]. The use of CNN is feasible for achieving automatic computer-based extraction of characteristics [23]. The ENVINet5 model is a semantic segmentation model that is suitable for the segmentation of simple and structure-fixed images. The model uses a few training images to create training datasets. The accuracy of segmentation is acceptable and has been widely applied for the recognition of biomedical images. The ENVINet5 model could be very feasible for the recognition of bovine distributions limited in structure and sample size [24]. Mask R-CNN can achieve pixel-level detection. For each target object, the bounding box is displayed and whether each pixel in the bounding box belongs to the object is marked [25]. The deep learning model is widely used in medical image diagnosis, face recognition, intelligent transportation, and other fields [26–28], but it is rarely used in monitoring the infectious source of schistosomiasis, in the present study, we explored to recognize livestock sources of the *S. japonicum* infection from high-resolution remote sensing images by using the ENVINet5 and Mask R-CNN models. The goal was to provide a technical basis for the monitoring and management of the source of *S. japonicum* infection.

## Methods

### Datasets

**Fig. 1:**
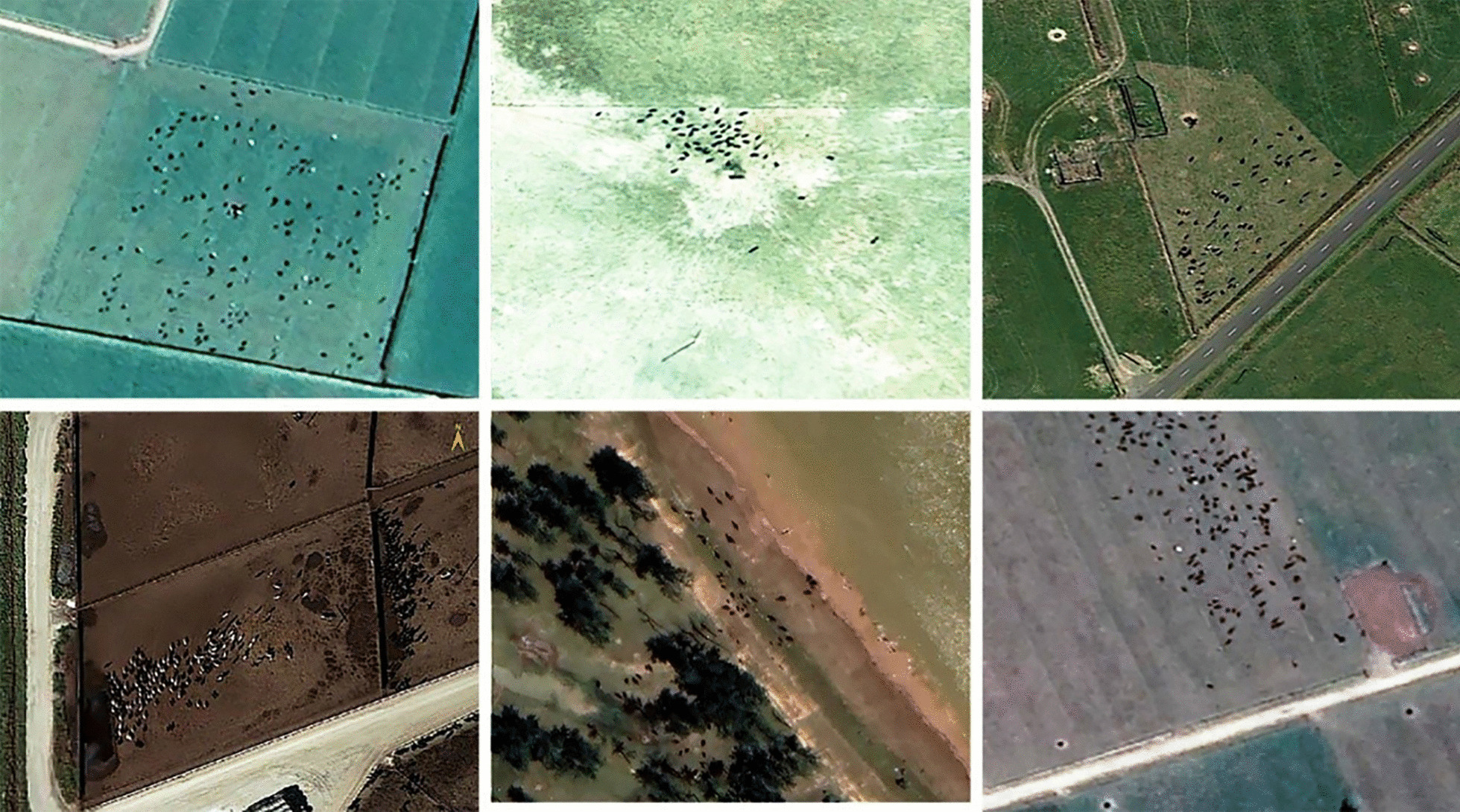
High-resolution images with bovine distributions

A dataset of high-resolution satellite images based on bovine distributions was collected from the Chinese National Platform for Common Geospatial Information Services (https://www.tianditu.gov.cn/). The image resolution was set as 0.3–0.4 m. The cloud coverage was < 5%. The images were selected based on different environmental characteristics and the equilibrium distribution of recognition objects. In addition, the sample images were selected based on multiple illumination conditions, multiple photography angles, and geographical areas. This image dataset spanned the years from 2016 through 2021. The dataset comprised a wide range of geomorphological types, including grasslands, mountains, and rivers. Based on these defined conditions, a total of 80 recognition objects were retrieved from the dataset (Fig. 1). They were classified into training and validation datasets and testing datasets for further model development, 70% of the entire dataset for training (Training data), 15% of the entire Dataset for validation (Validation data), 15% of the entire Dataset for testing (Testing data).

### Developing recognition models

Deep learning recognition models employ internal rules and indicative hierarchies along with computer-based image recognition to recognize and extract the target object from high-resolution images. The ENVINet5 model has a good performance when the training data is small, and the Mask R-CNN model has a good running speed and precision. In this study, deep learning recognition models to identify the spatial distribution of livestock were developed using the ENVINet5 and Mask R-CNN models in different scenarios.

### ENVINet5 model

The ENVINet5 model used the U-net network. The network is divided into two parts. The first is the contracting path that uses a typical CNN architecture. Each block in the contracting path consists of two successive 3 × 3 convolutions followed by a ReLU activation unit and a max-pooling layer. This arrangement is repeated several times. The novelty of U-net comes in the second part, called the expansive path, in which each stage upsamples the feature map using 2 × 2 up-convolution. Then, the feature map from the corresponding layer in the contracting path is cropped and concatenated onto the upsampled feature map. This is followed by two successive 3 × 3 convolutions and ReLU activation. At the final stage, an additional 1 × 1 convolution is applied to reduce the feature map to the required number of channels and produce the segmented image.

The energy function for the network is given by:

1 $$E=\sum w\left(x\right)\mathrm{log}{P}_{k\left(x\right)}(x)$$

where *w* is a weight map that we introduced to give some pixels more importance in the training, ${P}_{k}$ is the pixel-wise SoftMax function applied over the final feature map, defined as:

2 $${P}_{k}=\mathrm{exp}\left({a}_{k}\left(x\right)\right)/{\sum }_{{k}^{^{\prime}}=1}^{K}\mathrm{exp}({a}_{k}{(x)}^{^{\prime}})$$

In which ${a}_{k}$ denotes the activation in channel *K.*

### ENVINet5 model labeling

To capture the characteristic sample for developing image learning models, the recognized livestock should be first labeled for image datasets. The result is constructed as a label grid, which is used as an input for training deep learning recognition models. In the present study, the images were visually scanned for bovine before being sub-set into smaller areas, where we identified congregations of bovine.

To label training datasets in the ENVINet5 model, the typical areas were selected from the training dataset images for data creation and labeling. The typical area may fully represent the object characteristics of the entire image, and the object characteristics in the area of interest may partly indicate the characteristics of the whole image. The typical area is required to cover adequate target objects and may build adequate training datasets, thereby achieving better model training.

### Mask R-CNN model

Mask R-CNN is one of the methods of object detection and segmentation. It draws a bounding box for the target object and further marks and classifies whether the pixels in the bounding box belong to the object or not. These features can be used to identify and mark the boundary of the object and detect key points. Mask R-CNN is based on Faster R-CNN and extends its application to the field of image segmentation. The Mask R-CNN process uses the region proposal network (RPN) to extract features and to classify and tighten bounding boxes. Mask R-CNN replaces region of interest (ROI) pooling of Faster R-CNN with ROI alignment (RoIAlign), and consecutively uses the mask branch to mark the result of RoIAlign for the object area.

**Fig. 2:**
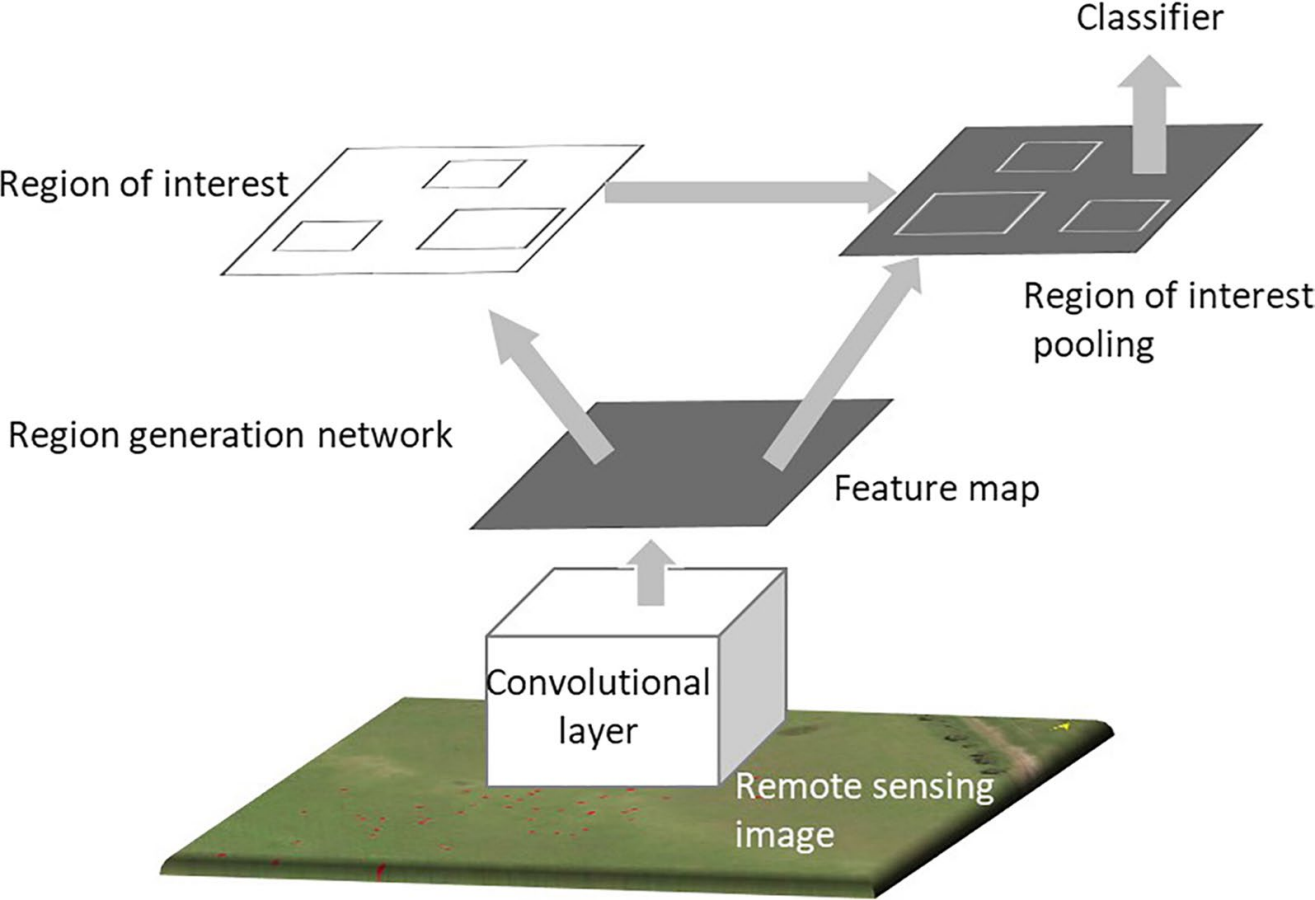
Mask R-CNN structure

The Mask R-CNN is a ResNet-based architecture, which allows the extraction of effective characteristics at a deeper network layer. Such a model is characterized by high calculation efficiency and recognition precision. In this study, the Mask R-CNN model was selected. The network flowchart is shown in Fig. 2.

In the RPN, the approximate regional framework of the target object was generated through characteristic maps combined with predesigned anchors. The characteristic region was screened using the Mask R-CNN architecture. The classification, border, and mask of each characteristic map were calculated using the network head. The overall network loss represented the sum of losses in classification, border, and mask. The mask loss (*L*_*mask*_) was calculated using the following formula:

3 $${L}_{mask}=-{\sum }_{i=1}^{N}{y}^{\left(i\right)}{\mathrm{log}\widehat{y}}^{\left(i\right)}+{\left(1-y\right)}^{\left(i\right)}$$

where *N* indicates the total sample number, ${y}^{\left(i\right)}$ indicates the expected output of Sample *i* (0 or 1), and ${\widehat{y}}^{\left(i\right)}$ indicates the actual output of Sample *i* (0 or 1) (i.e., the segmentation result). The learning rate and weight decay of the Mask R-CNN model were 0.001 and 0.0005, respectively. The optimal effect of the Mask R-CNN model was achieved following multiple-round training.

### Mask R-CNN model labeling

To capture the characteristic sample for developing image learning models, the recognized livestock should be first labeled for image datasets. The result is constructed as a label grid, which is used as an input for training deep learning recognition models. The images are visually scanned for bovine before sub-setting into smaller areas where congregations of bovines can be identified.

In the Mask R-CNN model, to ensure that training labels are representative of bovine at different times, images were selected for different seasons and years in both closed dense forests, grassland, and bare land. Images were labeled by defining bounding boxes around each individual bovine using the ‘Labelling’ graphical image annotation tool to increase the accuracy of bovine recognition. Data creation along the edge of bovine distribution was based on edge intensity monitoring results.

### Model validation

To evaluate the performance of the developed recognition models, the test dataset was used to validate the accuracy of deep learning models. The test images covered a wide range of geomorphological types, including grasslands, mountains, and rivers from different seasons and years.

In this study, to quantitatively evaluate the detection performance, performance metrics, including precision, recall, and F1-score, were used for algorithm evaluation. Recognition precision was defined as the ratio of the number of precisely recognized bovines in statistical images to the total number of target objects, which was calculated using the following formula:

4 $$P=\frac{TP}{TP+FP}$$

where *P* is precision, *TP* is a true positive (defined as the number of actual positives that are accurately identified) and *FP* is a false positive (defined as the number of negatives that are incorrectly identified as positives). The sum of *TP* and *FP* is the number of all identified positives and indicates the total number of recognized target objects in this study.

Precision is an indicator used to evaluate the performance of the predictive effect of a deep learning model. Recall (*R*) is defined as the proportion of actual positives that are accurately identified in all the original data. *R* was calculated using the following formula:

5 $$R=\frac{TP}{TP+FN}$$

where *FN* is the false negative, defined as the number of positives incorrectly identified as negatives. The sum of *TP* and *FN* is the total number of positives in the original data and represents the target objects in this study. Based on precision and recall, the F1-score is described as follows:

6 $$F1=\frac{2.P.R}{P+R}$$

### Field verification

Jiangxi Province is in the stage of schistosomiasis transmission control, by 2021, the number of livestock bovine in schistosomiasis endemic villages was 68,601 [12]. To further validate the performance of the developed models in the real world, we selected Houtian Township, Xinjian District, Nanchang City, Jiangxi Province as the field verification area. It is a schistosomiasis-endemic area. We applied the developed models for recognizing and monitoring the spatial distribution of bovines in a schistosomiasis-endemic area as the field validation.

## Results

### Discriminative features specificity of bovines

**Fig. 3:**
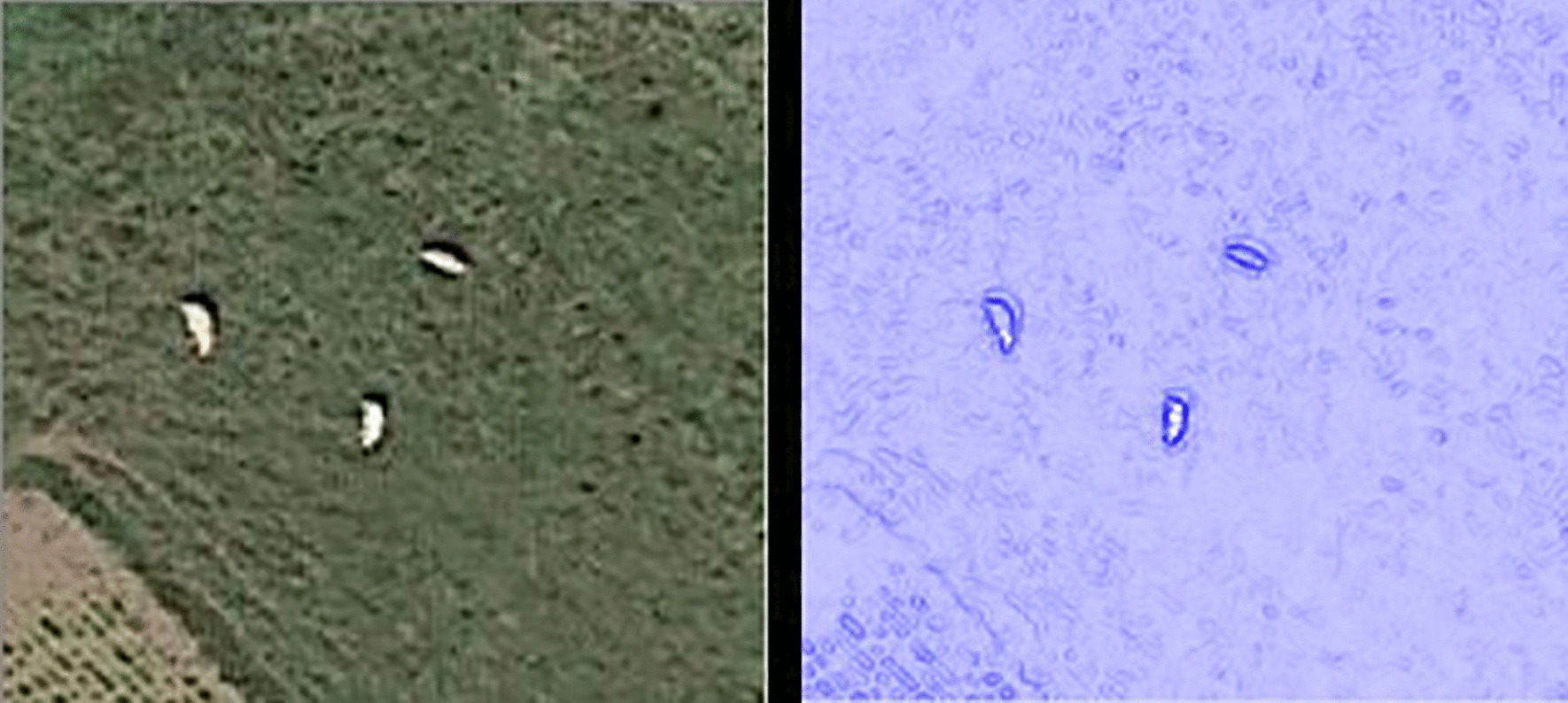
Characteristics of bovines in the images

Bovines identified by the developed recognition models for monitoring the source of *S. japonicum* infection had three characteristics. First, the size of recognized objects had a width of 6–7 pixels on images, which was equivalent to 2 or 3 m in reality. Second, the bovines were mainly found in grassland, bare land, island beaches, woodlands, and other areas. Third, the pixels where the bovines were located were obviously different from the surrounding background. Edge detection revealed that the edge intensity value of bovines was relatively large (Fig. 3).

### Results of bovine recognition

**Fig. 4:**
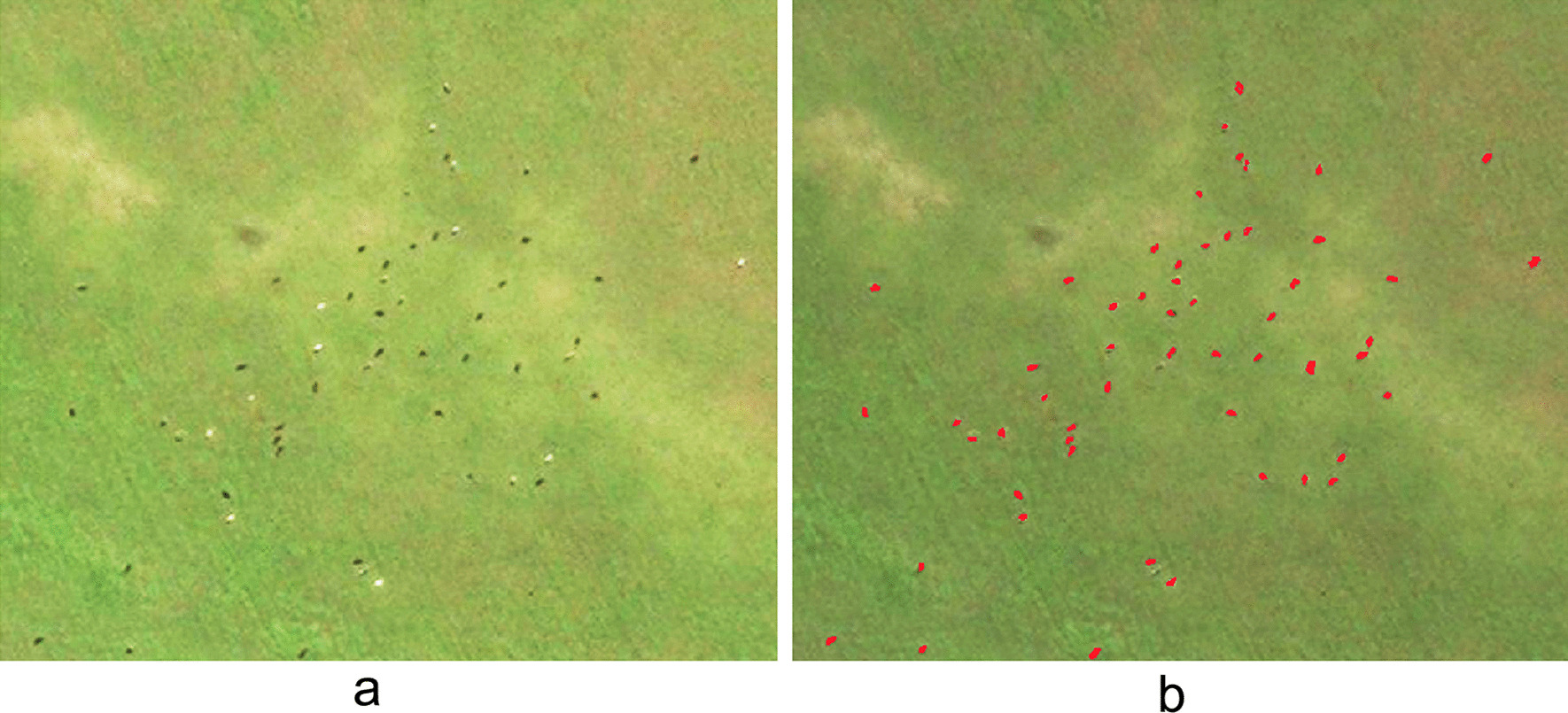
Sample labeling of the ENVINet5 model. **a** original images; **b** image labeling

In this study, a total of 50 images were selected, and 1125 bovine objectives were labeled for the ENVINet5 model. Figure 4 shows the labeling of selected typical images.

**Fig. 5:**
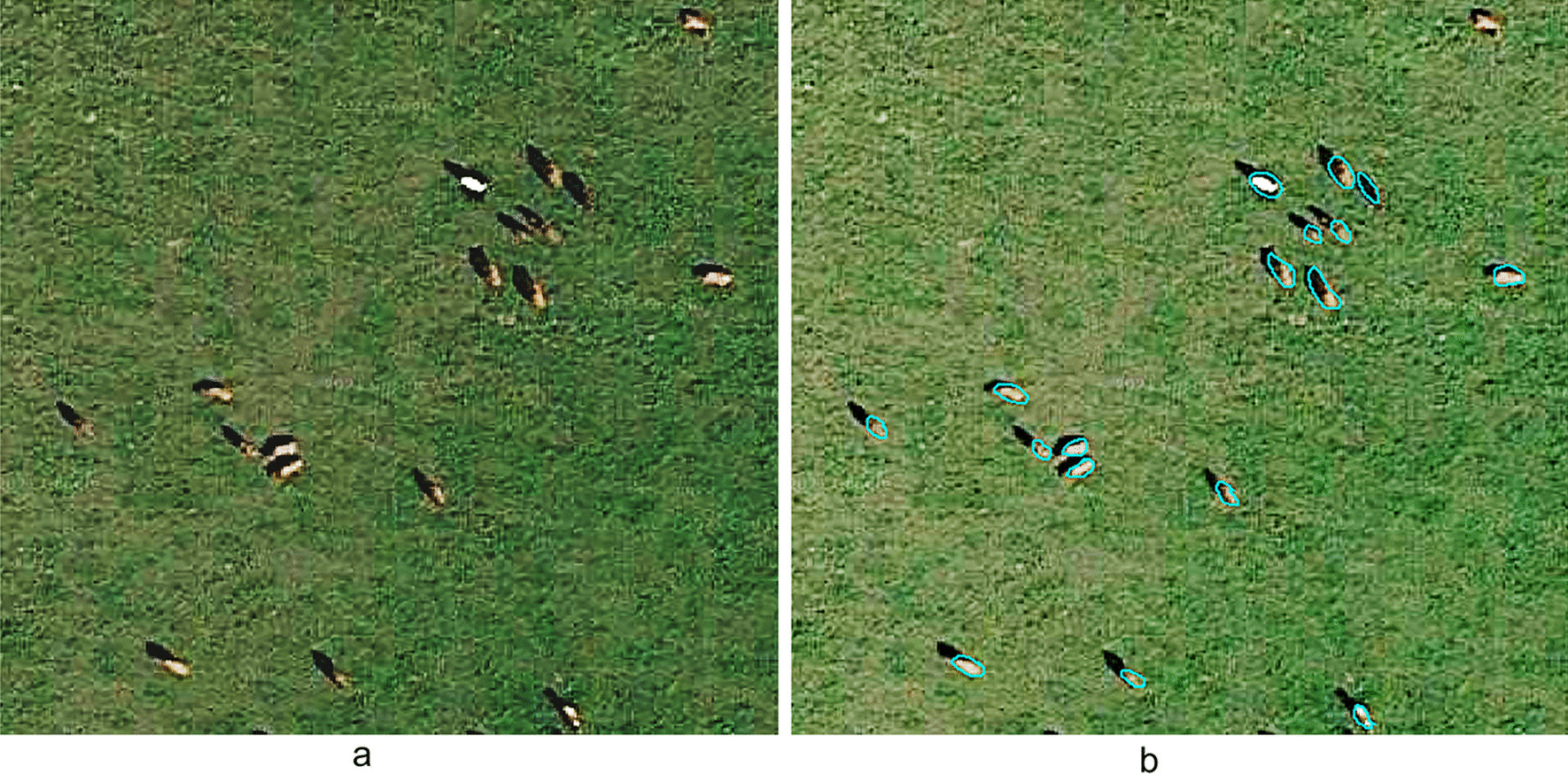
Sample labeling of the Mask R-CNN model. **a** original images; **b** image labeling

A total of 1277 training objectives were labeled with the Mask R-CNN model. Figure 5 shows the labeling of typical bovine objects.

We evaluated the efficacy of ENVINet5 and Mask R-CNN with the pre-labeled image dataset. In the ENVINet 5 model, parameters for patch size, number of epochs, number of patches per epoch, number of patches per batch, and patch sampling rate, were set as Table 1.

**Table 1:** The parameters of ENVINet5

Parameters	Patch size	Number of epochs	Number of patches per epoch	Number of patches per batch	Patch sampling rate
Value	425	20	400	16	5

In the Mask R-CNN model, the network hyper-parameters, including the momentum, learning rate, decay factor, training steps, and batch size were set as Table 2 through cross-validation. To better analyze the training process, we set up 100 epochs for training.

**Table 2:** The parameters of Mask R-CNNE

Parameters	Momentum	Learning rate	Decay factor	Training steps	Batch size
Value	0.9	0.001	0.0005	934	1

**Fig. 6:**
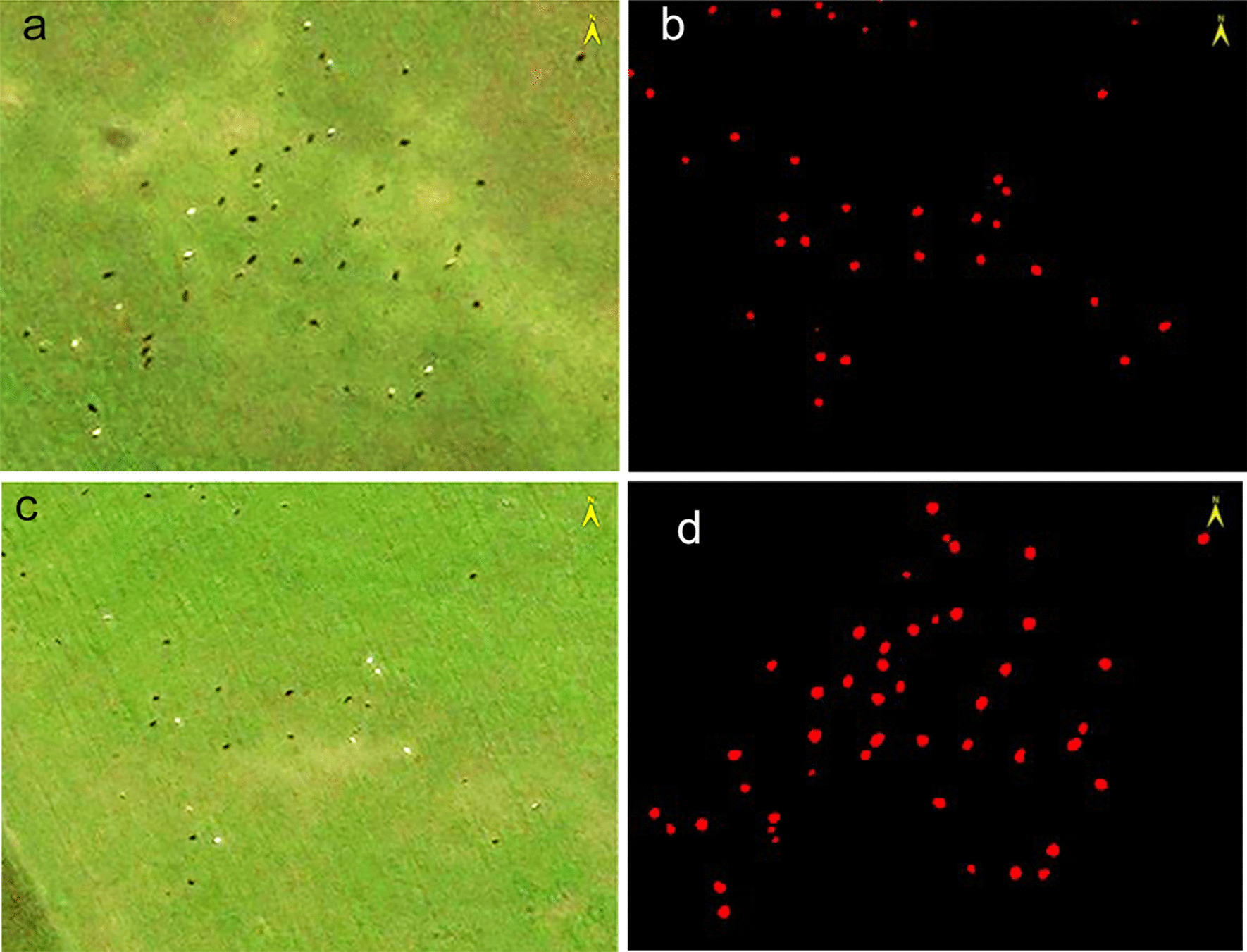
Extraction results of the ENVINet5 model. **a**, **c** is the original images. **b**, **d** is the result of extraction

**Fig. 7:**
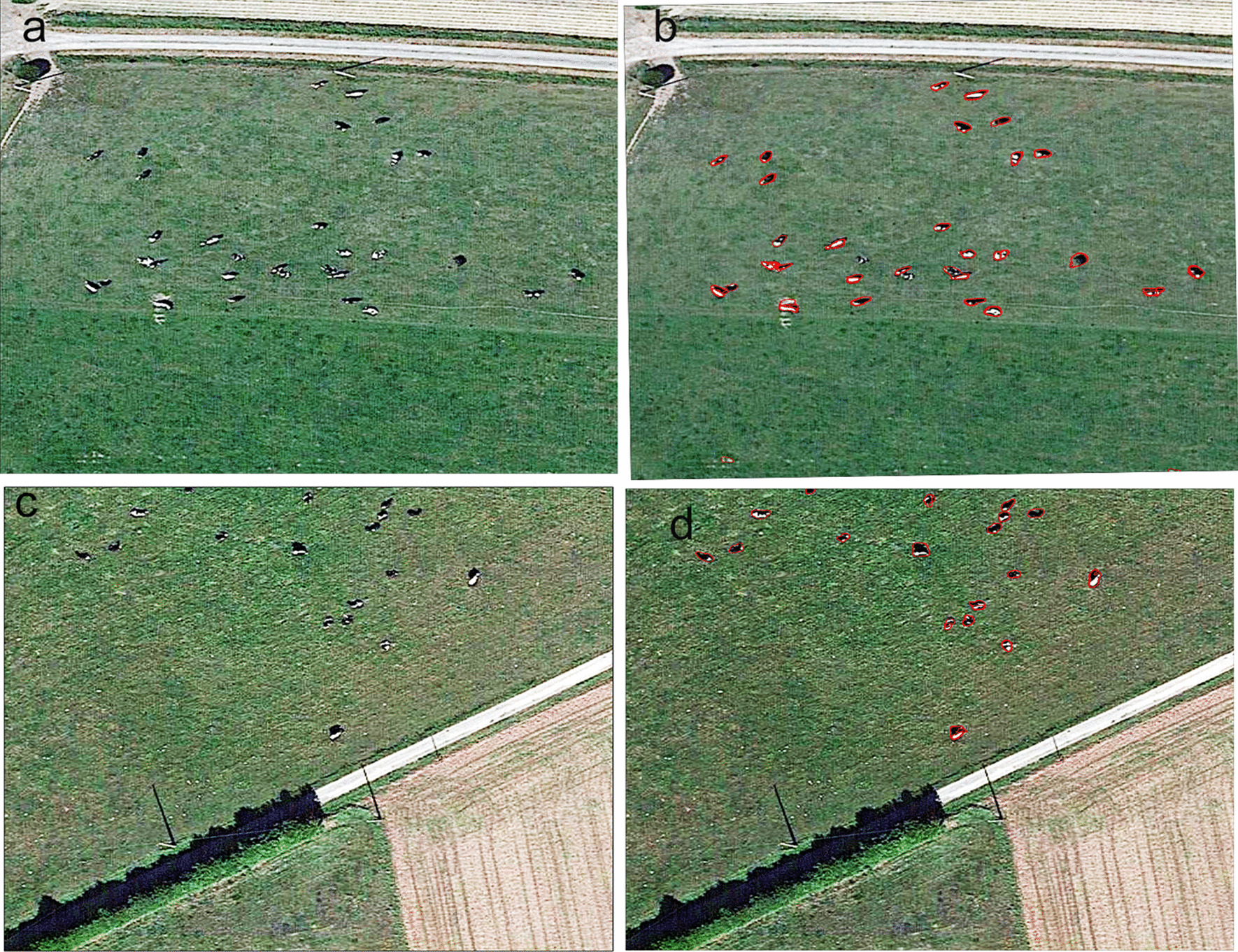
Extraction results of the Mask R-CNN model. **a**, **c** is the original images. **b**, **d** is the result of extraction

Using the above model parameters, we extracted target objects from the labeled datasets built by the ENVINet5 and Mask R-CNN models. The images of 80 selected objects of interest were extracted with the ENVINet5 model, and a total of 1598 target bovines were extracted. Figure 6 displays the extraction of selected objectives. In addition, a total of 1679 target bovines were extracted with the Mask R-CNN model. Figure 7 displays the regional extraction of four typical images.

### Model precision and recall

The developed ENVINet5 and the Mask R-CNN models were implemented with test datasets for model validation. A total of 310 test datasets were built. The precision and recall of the ENVINet5 and Mask R-CNN models were 81.9% and 80.2%, and 87.3% and 85.2%, respectively. The respective F1-scores were 0.81 and 0.87 (Table 3).

**Table 3:** Number of training and test datasets and test accuracy of models

Model	Number of training datasets	Number of test datasets	Accuracy		
			Precision (%)	Recall (%)	F1-score
ENVINet5	1125	320	81.9	80.2	0.81
Mask R-CNN	1277	320	87.3	85.2	0.87

The differences between ENVINet5 and Mask R-CNN models were obvious. Mask R-CNN had a better recall and precision, indicating that it can detect bovine more accurately. However, the model struggled to predict a good segmentation mask. Mask-RCNN had a relatively better performance compared to ENVINet5 for over- and under-segmentation.

### Field verification

**Fig. 8:**
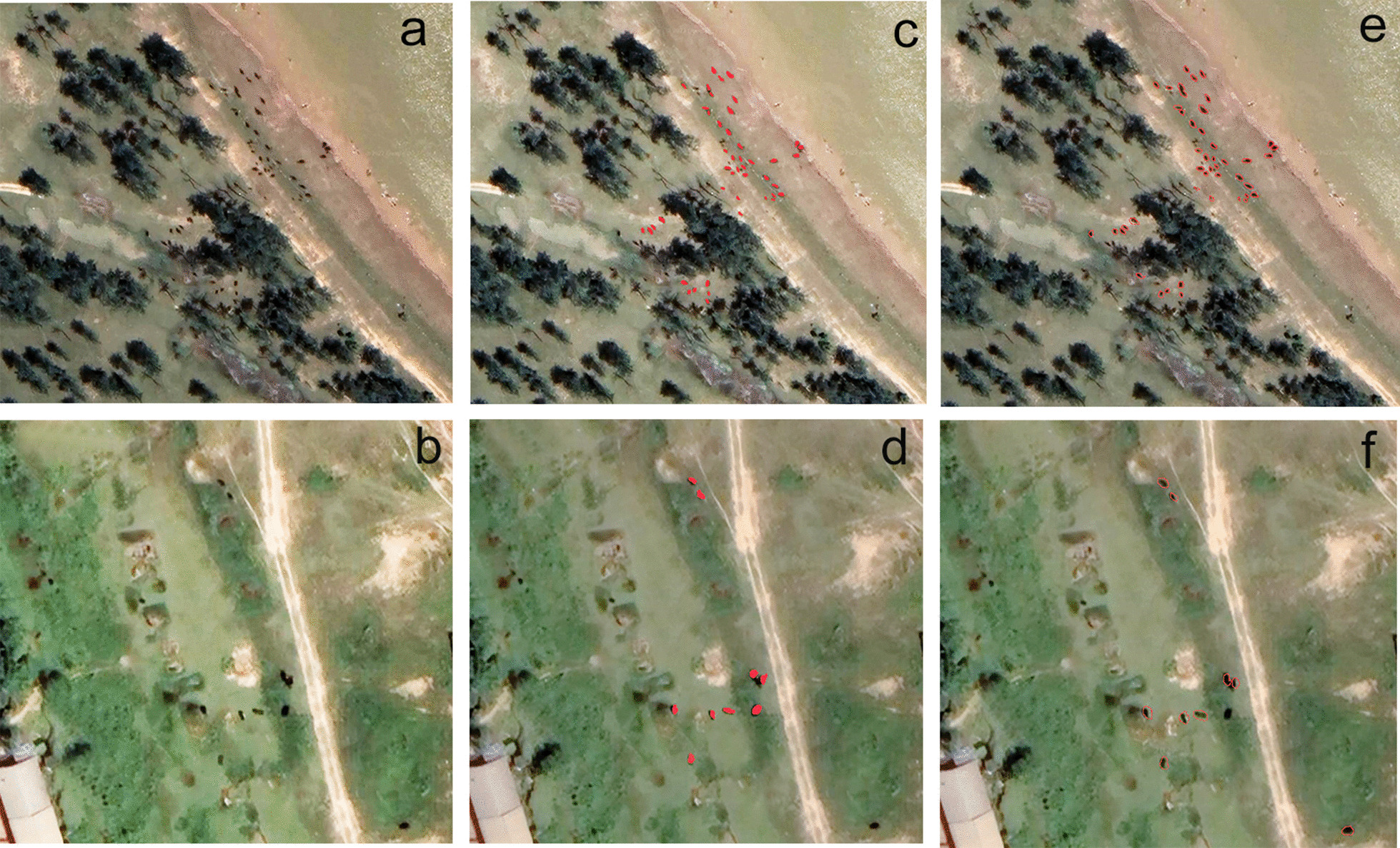
Results of the ENVINet5 model and Mask R-CNN model for extraction verification. **a**, **b** is the original remote images, **c**, **d** is the result of ENVINet5 extraction **e**, **f** is the result of MASK R-CNN extraction

As shown in Fig. 8, the spatial distribution of bovines was extracted from the image using the developed recognition models. There are 63 bovines in the original image. 53 were extracted using the ENVINet5 model and 57 using the Mask R-CNN model.

## Discussion

Schistosomiasis japonica remains a major public health concern in China. With concerted efforts for seven decades, great success has been achieved in schistosomiasis control [29, 30], and China is moving towards the elimination of schistosomiasis [31, 32]. Nevertheless, there are still many challenges for schistosomiasis elimination in China because the settings in schistosomiasis-endemic foci have not completely changed and there are widespread factors associated with schistosomiasis transmission [33, 34].

Previous studies have shown that bovines are critical in the transmission of schistosomiasis in China [35]. The residents of schistosomiasis-endemic foci have a habit of free pasturing in marshlands and grasslands. Because of repeated infection with *S. japonicum*, high parasite burdens in bovines, and high amounts of defecation, bovines reportedly contribute to most (80%) of the transmission of schistosomiasis [36]. The schistosomiasis transmission risk foci or *S. japonicum* infected, snail-infested sites that are identified in endemic foci are mostly attributed to the contamination of livestock stool [37]. Therefore, the management of livestock is effective in controlling the prevalence of schistosomiasis [38]. The implementation of an integrated strategy with an emphasis on controlling the source of *S. japonicum* infection has resulted in remarkable effects in the livestock schistosomiasis control program [39]. However, the prevalence of *S. japonicum* infection in livestock may be underestimated, and there are still problems in livestock schistosomiasis control programs that should be noted. For example, the prohibition of pasture in grasslands and building safe pastures are theoretically effective in some schistosomiasis-endemic areas. However, a lack of effective management makes it difficult to achieve the expected effects of schistosomiasis control [40].

The number of bovines in schistosomiasis-endemic foci in China has decreased dramatically since the integrated strategy was implemented, with an emphasis on eliminating the source of *S.* *japonicum* infection [41]. It is now difficult to detect the presence of bovines through manual investigations. In this study, we aimed to investigate the feasibility of high-resolution remote sensing image recognition for monitoring bovine distribution. In doing so, we utilized ENVINet5 and Mask R-CNN deep learning-based models. We observed 81.9% overall precision of the ENVINet5 model and 87.3% overall precision of the Mask R-CNN model for the extraction of bovine distribution data. The results suggest that high-resolution remote sensing image recognition combined with deep learning models is feasible for monitoring livestock in schistosomiasis-endemic foci in China.

The ENVINet5 model features high performance against interference, with no requirement for complete labeling. Therefore, this model is more convenient to build labeled datasets and remains effective to extract the target object in presence of a few labeled datasets [42]. The Mask R-CNN model needs to create labels along the target object and requires a long time for data training. However, the Mask R-CNN model features a good framework design and runs highly efficiently, which allows the effective detection of targets in images and generates a high-quality segmentation mask for each case [43]. In the present study, the Mask R-CNN model had better performance for the extraction of bovine distribution data than the ENVINet5 model.

Currently, China is striving to increase the early surveillance and forecast capability, improve the mechanism for surveillance of infectious disease epidemics and public health emergency, enhance the sensitivity and accuracy of assessment and monitoring, build a multi-touch mechanism for intelligent forecast, perfect multi-channel surveillance and forecast mechanisms, and improve the capability of real-time analysis and centralized determination [44]. Our study demonstrates the feasibility of deep learning for monitoring of livestock distributions in schistosomiasis-endemic foci of China, which provides a novel tool for schistosomiasis surveillance.

For the extraction of bovine distribution data, both the ENVINet5 and Mask R-CNN models had issues with false detection and missed detection. Further studies are required to improve the visualized effect of detections and the precision of target detections and optimize the framework of deep learning models for target detection. In addition, selection and improvements of appropriate deep learning models for the recognition of bovine distributions with high-resolution remote sensing images are needed for sensitive monitoring of livestock in schistosomiasis-endemic foci. The livestock schistosomiasis control program will facilitate animal husbandry developments and protect human health in schistosomiasis-endemic foci and accelerate the progress toward the elimination of schistosomiasis in China.

This study has several limitations. First, due to the limitation of remote image resolution, bovines appear as a width of only several pixels on sub-meter high-resolution remote sensing images. Their spectral and morphological characteristics can be easily confused with those of other mass-shaped targets, such as dark grasslands, vertical shadows of artificial buildings, vehicles, undershrub, and other animals [45, 46]. Second, the acquisition of high-resolution remote sensing images is difficult and expensive, which makes it impossible to continuously observe schistosomiasis endemic areas.

## Conclusions

With the increase in the resolution of remote sensing images and the decline in costs, high-resolution satellite images as a tool for surveying wild animals will become more popular in the future. The development of automatic detection tools is therefore of great value for the large-scale monitoring of wild animals. Two deep learning models, ENVINet5 and Mask R-CNN displayed good performance. It is thus possible to precisely control schistosomiasis by recognizing the spatial distribution of bovine using deep learning methods and high-resolution remote sensing images.

## Data Availability

The datasets used and/or analyzed during the current study are available from the corresponding author on reasonable request.
